# Thematic series: Epigenetics in stem cells and cancer

**DOI:** 10.1186/2045-3701-3-38

**Published:** 2013-10-09

**Authors:** Jing Huang

**Affiliations:** 1 National Cancer Institute, Bethesda, MD, USA

## Commentary

Epigenetics describes the inheritable changes in gene expression without DNA alteration, and its regulation is involved in stem cell differentiation, development, and tumorigenesis. This thematic series aims to summarize recent epigenetic studies in embryonic and adult stem cells, compare and contrast the epigenetic difference between stem cells and cancer cells, and raises provocative new concepts for cancer treatment.

Tumorigenesis and development are classically regarded as two separate processes. The classical definition of development describes the embryonic period between zygote and birth, while tumorigenesis happens after birth. However, many embryonic pathways are re-activated and/or dys-regulated in cancer cells. Therefore, studies of stem cell differentiation and development have provided and will continue to provide mechanistic insights into tumorigenesis. Embryonic stem (ES) cells are derived from blastocysts, and they have the ability to develop into the three germ layers, endoderm, mesoderm, and ectoderm. Because of the pluripotency, ES cells are a good model system to study epigenetics during stem cell differentiation and early development. After birth, adult stem cells are the repositories to maintain the homeostasis and to cope with stresses and injury of adult tissues. From the standpoint of cancer, ES cell differentiation is relevant to teratomas and testicular tumors. Interestingly, many types of carcinomas share overlapping transcriptomes with ES cells, suggesting that part of the embryonic transcription program is re-activated in tumors, possibly via epigenetic regulation [1,2]. Accumulating evidence indicates that many types of tumors consist of stem cell-like cells, dubbed cancer stem cells (CSCs) or cancer initiating cells (CICs). CSCs or CICs may come from transformed adult stem cells or terminally differentiated cells that are reprogrammed. To fully understand the epigenetic alterations in cancer stem cells, it is important to have a global picture of the epigenetic landscapes in normal adult stem cells.

Compared with adult stem cells and somatic cells, ES cells have a distinct epigenetic landscape [3]. How that landscape affects ES cell differentiation and pluripotency has attracted a lot of attention. Gu et al., review the roles of one of the most well studied histone methylations, histone H3 lysine 4 methylation (H3K4me), in ES cells as well as in adult stem cells [4]. Because many of the enzymes that regulate H3K4me are also involved in tumorigenesis, this review may advance our understanding of H3K4me-modifying enzymes in cancer.

Myogenesis is the process of the development of heart and skeletal muscles. It is one of the most well documented differentiation processes of ES cells. Abnormal myogenesis may be also related to rhabdomyosarcomagenesis. Chen and Li summarize the epigenetic events and signaling pathways that regulate myogenesis from ES cells [5]. They concentrate on histone acetylation and small molecule compounds that can induce myogenesis.

Certain types of tumors have CSCs or CICs, which have similar features as adult stem cells. Tarayrah et al., systematically review the epigenetic regulation of several adult stem cells, e.g. germline stem cells (GSCs), intestinal stem cells (ISCs), and hair follicle stem cells [6]. In addition, abnormal epigenetic regulations in cancer cells, such as DNA methylation, histone modifications, and chromatin remodeling, are summarized. Because epigenetic changes are reversible, they represent attractive intervention nodes for cancer treatment.

The tumor suppressor p53 plays many important roles in regulating stem cell differentiation and suppressing tumors. Shin et al., highlight the recent studies of p53 in ES cells, and describe how these studies provide mechanistic insights into the relationship between the developmental role and tumor suppressive function of p53 [7].

## Competing interests

The authors declare that they have no competing interests.
